# Hyperoxia Induced Bronchopulmonary Dysplasia-Like Inflammation *via* miR34a-TNIP2-IL-1β Pathway

**DOI:** 10.3389/fped.2022.805860

**Published:** 2022-03-30

**Authors:** Xuwei Tao, Luxia Mo, Lingkong Zeng

**Affiliations:** Department of Neonatology, Wuhan Children’s Hospital (Wuhan Maternal and Child Healthcare Hospital), Tongji Medical College, Huazhong University of Science and Technology, Wuhan, China

**Keywords:** miR34a, BPD (bronchopulmonary dysplasia), inflammation, interleukin-1β (IL-1β), TNIP2

## Abstract

Lung injury induced by oxygen is a key contributor to the pathogenesis of preterm infant bronchopulmonary dysplasia (BPD). To date, there are comprehensive therapeutic strategy for this disease, but the underlying mechanism is still in progress. By using lentivirus, we constructed microRNA34a (miR34a)-overexpressing or knockdown A549 cell lines, and exposure to hyperoxia to mimic oxygen induce lung injury. In this study, we investigated 4 proinflammatory cytokines, interleukin-1β (IL-1β), tumor necrosis factor-α (TNF-α), angiopoietin-1 (Ang-1), and Cyclooxygenase-2 (COX-2) in the secreted sputum of infants who received mechanical ventilation, and found that IL-1β was substantially elevated in the first week after oxygen therapy and with no significant decrease until the fourth week, while TNF-α, Ang-1, and COX-2 were increased in the first week but decreased quickly in the following weeks. In addition, *in vitro* assay revealed that hyperoxia significantly increased the expression of miR-34a, which positively regulated the proinflammatory cytokine IL-1β in a time- and concentration-dependent manner in A549 cells. Overexpressing or knockdown miR34 would exacerbate or inhibit production of IL-1β and its upstream NOD-, LRR-, and pyrin domain-containing protein 3 (NLRP3) inflammasome signaling pathway. Mechanically, it’s found that TNFAIP3 interacting protein 2 (TNIP2), an inhibitor of nuclear factor κB (NF-κB), is a direct target of miR34a, negatively regulated activation of NLRP3 inflammasome and the production of IL-1β. Overexpressing TNIP2 ameliorated hyperoxia-induced production of IL-1β and cell apoptosis. Our findings suggest that TNIP2 may be a potential clinical marker in the diagnosis of BPD.

## Introduction

Bronchopulmonary dysplasia (BPD), defined as the need for supplemental oxygen after 36 weeks post-menstrual age, affects around 30% of infants with a birth weight less than 1,000 g (1). BPD is a leading cause of long-term hospitalization, stunted growth, recurrent respiratory illness, and neonatal death (2). Abnormal lung function and structures associated with BPD persist into adolescence (3, 4). Until now, there is no particular therapy or therapeutic agent accessible to relieve BPD, since many factors that can contribute to the development of BPD, such as genetics, oxygen damage, lung damage, infection, and malnutrition, etc. (5). MicroRNAs (miRNA) are single-stranded and evolutionarily conserved sequences of short non-coding RNAs (∼21–25 nucleotides), which act as endogenous repressors of gene expression by mRNA degradation and translational repression (6, 7), and have been demonstrated to have critical roles in cell differentiation, development, proliferation, signaling, inflammation, and cell death (8, 9). Hence, they are considered to be the promising candidates for novel targeted therapeutic approaches to lung diseases. The role of oxygen in the development of BPD has been evaluated in a few studies, which explored the expression profiles of miRNA in various animal models and human infants (10). MiR-34a expression was significantly increased after oxygen exposure, peaked at postnatal day (PN) 7, and then steadily declined (11). It has been reported that neonates with respiratory distress syndrome and BPD have shown an increased miR-34a expression in type 2 alveolar epithelial cells, suggesting that miR34a might be a potential pharmacologic target (11). As the loss or gain of function of miR-34a improves or aggravates BPD-associated pulmonary arterial hypertension in BPD mouse models, its role in BPD still needs to be explored.

Interleukin-1β (IL-1β), a central cytokine involved in the initiation and persistence of inflammation, is increased in amniotic fluid in chorioamnionitis and preterm labor (12). Increased IL-1β concentration in amniotic fluid has been verified to be linked to the development of BPD (13). IL-1β levels were also elevated postnatally in the tracheal aspirates of preterm infants who develop BPD (14). A bi-transgenic mouse with conditional IL-1β expression in airway epithelial cells developed a series of lung diseases that were clinically and histologically comparable to BPD (15). Oxygen therapy caused pulmonary inflammation, and oxygen exposure enhanced the production of neutrophil chemo-attractants, resulting in infiltration of the lungs with neutrophils (16). NOD-, LRR-, and pyrin domain-containing protein 3 (NLRP3) inflammasome and its downstream Caspase 1, regulated the production of inflammatory molecules such as IL-1β (17), and miR-34a suppression reduces lung inflammation and apoptosis in an LPS-induced acute lung damage mouse model, indicating that miR-34a may mediate inflammasome activation (18). Except IL-1β, miRNA-34a also up-regulates TNF-α, IL-6, and Ang-1 production in animal or cell model (11, 19–21), which all are proinflammatory factors that closed relate with progress of BPD. However, the dynamics of these pro-inflammatory cytokines during the development of BPD in preterm infants and their role in BPD have not been reported in detail. And there is still no experimental evidence of how miR34a regulates NLRP3 inflammasome and the expression of cytokines.

The focus of this research was to analyze the expression of miR-34a in airway epithelial cells and the secretion of IL-1β and its upstream components. The difference in the expression of the above factors at different time points after birth can aid in developing early monitoring and early warning of BPD, and a prognostic judgment to provide monitoring signs. Moreover, this study tried to explore the mechanism during which to target the impact of miR-34a expression changes on downstream factors.

## Materials and Methods

### Establishment of A549 Cell Lines and Hyperoxia Exposure

Human alveolar basal epithelial cells A549 cells were purchased from China Center for Type Culture Collection (CCTCC, Wuhan, China), and were maintained in Dulbecco’s Modified Eagle Medium (DMEM) supplemented with 10% Fetal Bovine Serum (FBS), 100 U/ml penicillin, 100 μg/ml streptomycin (GIBCO, CA, United States) at 37°C in 95% air, 5% CO2. miR34a overexpression or knockdown A549 cell lines were generated using pBABE-puro-miR34a or pBABE-puro-si-miR34a lentivirus and packaging cell line A549, as previously described (22). Infected A549 cells were selected by adding puromycin (2 μg/mL) 3 days after infection and overexpression or knockdown efficiencies were examined by GFP fluorescence and identified by sequencing. For hyperoxia experiments, cells at sub-confluence (70%, ∼350–400 cells/mm^2^) were placed in sealed glass chambers filled with 95% O_2_–5% CO_2_, 60% O_2_–5% CO_2_, 35% O_2_–5% CO_2_, at 37°C, and culture for 24, 48, or 72 h, respectively. Normoxic cells were kept in normal air conditions (21% O_2_–5% CO_2_) at 37°C. Mediums and gases were replaced every 2 days.

### Western Blotting Analysis

After hyperoxia exposure, cell proteins were extracted as previously described (23). Proteins were blotted on a nitrocellulose membrane and then incubated with antibodies. The membranes were then washed and incubated with a horseradish peroxidase-conjugated anti-mouse antibody or peroxidase-conjugated anti-rabbit antibody (1:1,000; Beyotime, Shanghai, China). Proteins were detected by using ECL reagents (Biosharp, Beijing, China). Densitometric evaluation was performed using Image J (NIH, United States).

### Preterm Infant Criteria and Sputum Collection

A total of 37 infants who finally diagnosed as BPD hospitalized to the Neonatal Intensive Care Unit of Wuhan Children’s Hospital between January 2021 and September 2021 were recruited. Inclusion criteria: (1) Gestational age ≤ 32 weeks; (2) The legal guardians of the infants agreed and signed the informed consent form. Exclusion criteria: (1) Infants whose families abandoned treatment during hospitalization or whose families withdrew midway; (2) Infants with complex congenital heart disease, respiratory malformations, congenital genetic metabolic diseases, and those requiring surgical intervention such as neonatal necrotizing enterocolitis and diaphragmatic hernia, etc. Detail clinical characteristics of infants were listed in Table 1. According to the diagnostic criteria (24), these infants were divided into 3 groups, 5 cases were severe BPD, 11 cases were moderate BPD, and 21 were mild BPD. As control, other 95 non-BPD infants were enrolled. In 24 h after birth, 0.5 ml of respiratory secretions were retained by deep aspiration and tested within 24 h. Sputum was extracted *via* tracheal intubation in intubated patients and deep aspiration by suction tube in non-intubated patients. Aspiration operation was then performed once a week. All parents of infants have been aware of and provide written informed consent for their participation, and have also obtained permission from the Ethics Committee of Wuhan Children’s Hospital (2021R050-E01), Tongji medical college, Huazhong University of Science and Technology, Wuhan, 430077, China.

**TABLE 1:** Clinical characteristic of BPD infants.

	BPD (*n* = 37)	Non-BPD (*n* = 95)	*P* value
Gender (male, %)	20 (54.05%)	53 (55.79%)	0.67^a^
Gestational age (Weeks, $\bar{x}$ s)	28.63 ± 1.57	31.09 ± 0.74	<0.01^b^
Weight (g, $\bar{x}$ s)	1244.67 ± 241.36	1690.92 ± 235.86	<0.01^b^
Fetal distress (%)	9 (24.32%)	36 (37.90%)	0.37^a^
Apgar-1 [score, M (P25, P75)]	6 (4, 7)	8 (7, 8)	<0.01^c^
Apgar-5 [score, M (P25, P75)]	7 (5, 8)	9 (8, 9)	<0.01^c^
Apgar-10 [score, M (P25, P75)]	8 (5, 8)	8 (8, 9)	<0.01^c^
Time of PS used (more than once, %)	10 (27.03%)	2 (2.11%)	<0.01^a^
Invasive mechanical ventilation used (days, $\bar{x}$ s)	10.12 ± 16.09	0.94 ± 0.46	<0.01^b^
CPAP used (days, $\bar{x}$ s)	27.24 ± 10.92	15.89 ± 6.22	<0.01^b^
length of stay (days, $\bar{x}$ s)	57.82 ± 16.21	30.62 ± 12.32	<0.01^b^
Early sepsis (%)	6 (16.22%)	12 (12.63%)	0.54^a^
Prenatal glucocorticoid Administration (%)	7 (18.92%)	12 (12.63%)	0.25^a^
Maternal Chorioamnionitis (%)	5 (13.51%)	1 (1.05%)	<0.01^a^
Cesarean section (%)	13 (35.14%)	47 (49.47%)	0.36^a^

*^a^Chi-square test, Fisher’s exact probability method was used for n < 40.*

*^b^Independent sample t-test.*

*^c^Two-Sample K-S Test.*

### Enzyme Linked Immunosorbent Assay

Part of the sputum was weighted and add 0.1% DTT (Dithiotreitol) twice as much as the volume of the sputum. The sputum was blown repeatedly with a straw, and vortex for 15 s, and vibrated in a 37°C water bath for 5 min. Then add PBS buffer twice the amount of sputum and continued to vibrate for 15–20 min, then filter with 150 pieces of wire mesh, centrifuge at 1500 rpm for 10 min and extract the supernatant for detection. Concentrations of cytokines (IL-1β, TNF-α, Ang-1, and COX-2) were measured according to the manufacturer’s instructions. The levels of IL-1β, TNF-α, and Ang-1 in the sputum of infants were measured by enzyme linked immunosorbent assay (ELISA) kit from Elabscience (Wuhan, China). The COX-2 levels were determined using a human COX-2 ELISA kit from CUSABIO (Wuhan, China).

### Terminal Deoxynucleotidyl Transferase-Mediated dUTP Nick End Labeling Assay

Terminal deoxynucleotidyl transferase (TdT)-mediated dUTP nick end labeling (TUNEL) assay was performed on A549 cells grown on glass coverslips using One-step TUNEL Assay Kit (Red, AF594) (E-CK-A322, Elabscience, Wuhan, China) following manufacturer’s instructions. Quantification of TUNEL-positive cells was performed in selected images by an observer masked to the identity of the experimental groups. Cell density about 350–400 cells/mm^2^.

### Real-Time Quantitative Polymerase Chain Reaction

The total RNA was extracted by a TRIzol reagent (Invitrogen, CA, United States) following the manufacturer’s instructions. 1 μg of RNA was reversely transcripted for mRNA by a First-Strand cDNA Synthesis Kit (Yeasen, Shanghai, China) or a miRcute Plus miRNA First-Strand cDNA Kit (Tiangen, Beijing, China), respectively. The standard quantitative polymerase chain reaction (qPCR) was performed on an ABI StepOnePlus real-time quantitative PCR instrument using SYBR Green Mix (Yeasen, Shanghai, China). The reaction was performed with pre-denaturation at 95°C for 3 min, followed by 40 cycles of denaturation at 95°C for 5 s and annealing at 60°C for 30 s. This cycle was followed by a melting curve analysis, ranging from 60 to 95°C with temperature increase by steps of 0.5°C every 10 s. The method for miR34a’ reverse transcription referred to published literature (25). The primers used for RT-PCR detection were listed as following. miR34a-F: GGCAGTGTCTTAG CTGGTTG. miR34a-R: CCAGTGCAGGGTCCGAGGTATTC. miR34a-RT: GTCGTATCCAGTGCAGGGTCCGAGGTATTCGCACTGGAT ACGA CCAACCA. U6-F: GTGCTCGCTTCGGCAGCACATA. U6-R: GCGCAGGGGCCA TGCTAATCTTC. U6-RT: AAAAAT ATGGAACGCTTCACGAATTTG. IL-1β-F: ATGATGGCTTAT TACAGTGGCAA. IL-1β-R: GTCGGAGATTCGTAGCTGGA. TNIP2-F: AAGTCCTGACCAGTCGGAACA. TNIP2-R: TCTTC AACGTGAGTCA CCTTCT. ACTB-F: CATGTACGTTGCTA TCCAGGC. ACTB-R: CTCCTTAATGTC ACGCACGAT.

### Luciferase Activity Assay

The wild-type (WT) or mutant TNIP2 3’-untranslated region (UTR) plasmid was cloned and inserted into psiCHECK-2 within *Xho*I and *Not*I restriction sites located downstream of the Renilla luciferase gene. Wild-type TNIP2 3’-UTR sequence: GCUGGGUCACAGGGAACUGCCAG; mutant TNIP2 3’-UTR: GCUGGGUCACA GGGAGCUUCAAG. These plasmids were co-transfected into HEK293T cells with miR-34a mimic or scramble at a final concentration of 100 nM. After 48 h, cells were harvested, and lysates were used for firefly and Renilla luciferase activities using the dual-luciferase reporter assay kit (Promega, CA, United States) according to the manufacturer’s instructions. The normalized values (Renilla/firefly activity) were used for analysis. Experiments were performed in triplicate.

### Statistical Analysis

All data are expressed as Mean ± SEM. Unpaired *t*-test was used to assess statistical significance between the two groups. With respect to multiple comparisons involving three or more groups, statistical significance was assessed by one-way analysis of variance (ANOVA) followed by *post hoc* test (Bonferroni’s method). Statistics were computed with Graphpad Prism 6 (GraphPad Software). *P* < 0.05 was considered as statistically significant.

## Results

### Interleukin-1β Has Markedly Enhanced in Neonates Receiving Oxygen Therapy

**FIGURE 1:**
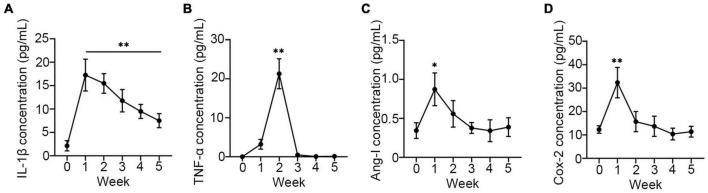
Proinflammatory was upregulated in sputum of neonates. The concentration of IL-1β **(A)**, TNF-α **(B)**, Ang-1 **(C)**, and COX-2 **(D)** were assay using ELISA in sputum of neonates every week after receiving oxygen therapy. One-way ANOVA, * *p* < 0.05, ^**^ *p* < 0.01 vs. 0 week, *n* = 37.

Although several proinflammatory cytokines were increased in infants following oxygen therapy (26), none of them have been implicated in the development of BPD. To investigate the expression of proinflammatory in the development of BPD, the secreted sputum of infants was collected and examined. As expected, all proinflammatory cytokines, including IL-1β, TNF-α, Ang-1, and COX-2 were markedly enhanced in the first week after oxygen therapy. However, except for IL-1β (Figure 1A), other cytokines (Figures 1B–D) returned to normal levels by the second week, while IL-1β remained elevated until the fourth week. And those infants with higher IL-1β concentrations eventually developed BPD. It was suggested that IL-1β may contribute more than others to the development of BPD.

### Expression of miR-34a and Interleukin-1β in Bronchopulmonary Dysplasia-Like Cell Model

**FIGURE 2:**
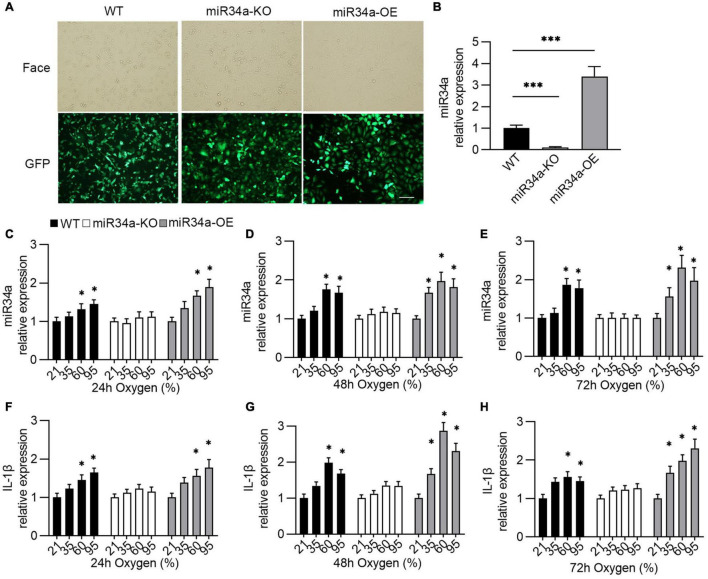
Establishment of oxygen-injury A549 cell model. **(A,B)** A549 cell that overexpression or knockdown of miR34a was established by being infected with lentivirus that carrying miR34a sequence or miR34a shRNA and their mRNA level **(B)**. One-way ANOVA, ^***^ *p* < 0.001 vs. WT, *n* = 5. Transfected cells images under the light (Face) and fluorescence (GFP) microscope. **(C–E)** The expression of miR34a was detected by qPCR after exposure to oxygen. **(F–H)** The expression of IL-1β was assayed using ELISA after exposure to oxygen. One-way ANOVA, * *p* < 0.05, ^***^ *p* < 0.001 vs. 21%, *n* = 5.

Previous research has linked miR34a to the development of BPD, and it also promotes the production of IL-1β. However, the underlying mechanism has not been explored. Thus, in this study, a BPD-like cell model using A549 cells (27, 28) was established by the overexpression (miR34a-OE) or knockdown (miR34a-KO) of miR34a in A549 cells (Figures 2A,B) to investigate the relationship between them. To mimic the situation of oxygen therapy in infants, wild type (WT), miR34a-OE, and miR34a-KO A549 cells were exposed to oxygen with different concentrations and times. It was observed that miR-34a expression increased in a time- and concentration-dependent way after oxygen exposure (Figures 2C–E). On the other hand, it was found that the mRNA level of IL-1β also increased in concentration- (35, 60, and 95%) and time- (24, 48, and 72 h) dependent ways (Figures 2F–H). The above results indicated that the production of IL-1β might has a closed with miR-34a.

### A549 Cell Apoptosis Was Mediated by miR34a

**FIGURE 3:**
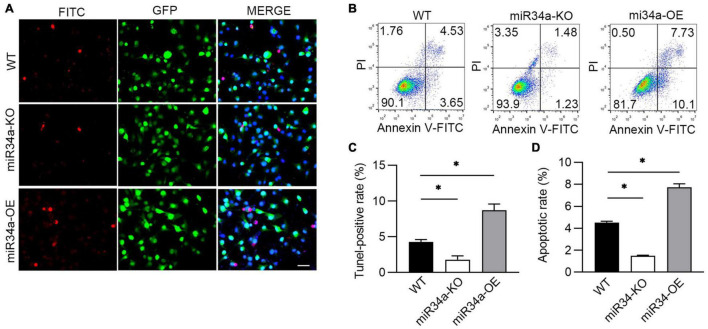
miR34a promoted A549 apoptosis induced by hyperoxia. **(A)** WT, miR34a-OE, and miR34a-KO A549 cell was staining by TUNEL-FITC (Red) after culture in slice and treated with hypoxia, and **(C)** its statistical result. **(B)** WT, miR34a-OE, and miR34a-KO A549 cell apoptotic rate was examined by flow cytometry and its statistical result **(D)**. One-way ANOVA, * *p* < 0.05 vs. WT, *n* = 5.

Proinflammatory cytokines cause injury to alveolar epithelial cells and lead to the development of BPD *via* inducing cell apoptosis (29). As BPD is characterized by inflammation and apoptosis of various cell types, we next investigated whether miR34a was involved in hyperoxia-induced apoptosis. The cells apoptotic rate in WT, miR34a-OE, and miR34a-KO A549 was checked by TUNEL-staining and flow cytometry. More apoptotic cells were observed in miR34a-OE A549 cells (Figures 3A,C), while markedly reduced in miR34a-KO A549 when compared to WT A549 cells. Flow cytometry (Figures 3B,D) confirmed that miR34a-KO ameliorated cell apoptosis induced by hyperoxia, while in contrast enhanced apoptotic rate greatly in miR34a-OE cell, suggesting miR34a mediated apoptosis in A549 cells.

### miR34a Mediated Hyperoxia-Activated NLRP3 Inflammasome Pathway

**FIGURE 4:**
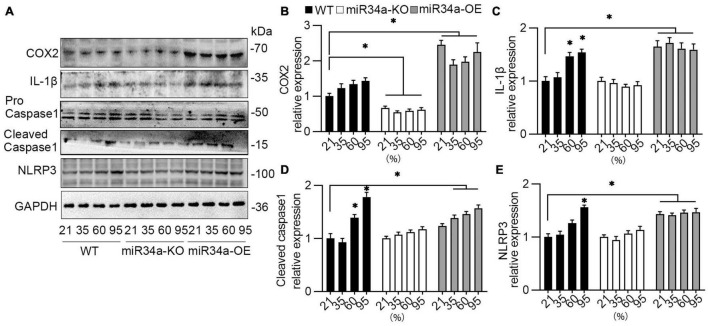
NLRP3 inflammasome pathway was activated by hyperoxia. **(A)** WT, miR34a-KO, and miR34a-OE A549 cells were collected after to different concentration of oxygen for 72 h, then examined by COX-2, IL-1β, caspase 1, and NRLP3 antibody using Western blotting and **(B–E)** their statistical results. One-way ANOVA, * *p* < 0.05 vs. 21%, *n* = 5.

One major pathway of IL-1β synthesis depends on the activation of the NOD-, LRR-, and pyrin domain-containing protein 3 (NLRP3) inflammasome, thus the expression of an NRLP3-related protein was detected. After exposure to different concentrations of oxygen for 72 h, A549 cells were collected and assayed using Western blotting. The expression of IL-1β, cleaved caspase 1, and NLRP3 were enhanced in a concentration-dependent way. However, knockdown miR34a prevented these increases, even with the induction of oxygen, while the overexpression of miR34a would further strengthen this enhancement (Figures 4A,C–E). Another proinflammatory cytokine COX-2, inducted by IL-1β, was also upregulated or blocked by overexpression or knockdown of miR34a (Figures 4A,B).

### Bioinformatic Analysis of miR34a’s Binding to 3’UTR of TNIP2

**FIGURE 5:**
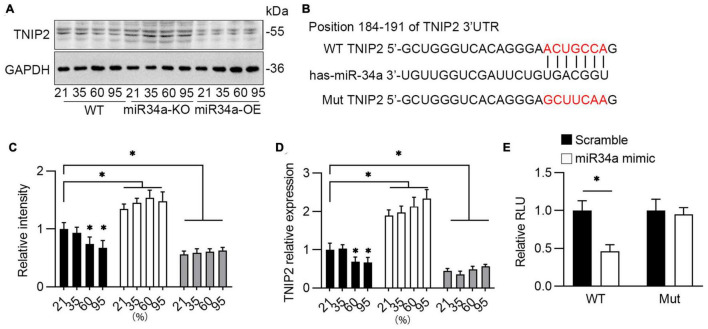
Bioinformatics analysis miR34a’s downstream target. **(A)** WT, miR34a-KO, and miR34a-OE A549 cells was collected after to different concentration of oxygen for 72 h, then examined by TNIP2 antibody using Western blotting and **(C)** its statistical results, One-way ANOVA, * *p* < 0.05 vs. 21%, *n* = 3. **(B)** Schematic diagram revealed bioinformatic prediction of miR-34a and 3’UTR of TNIP2 and mutated sequence. **(D)** qPCR analyzed mRNA of TNIP2 in WT, miR34a-KO, and miR34a-OE A549 cells after exposure to different concentration of oxygen for 72 h. One-way ANOVA, * *p* < 0.05 vs. 21%, *n* = 3. **(E)** HEK293T were transfected with pGL3-3’UTR TNIP2 and miR34a mimic or scramble for 24 h and then analyzed using dual luciferase reporter gene. Unpaired *t*-test, * *p* < 0.05 vs. scramble, *n* = 3.

As predicted by Targetscan,^1^ one target of miR-34a is TNIP2, a hub protein that can both positively and negatively regulate NF-κB-dependent transcription of target genes. It has been revealed that TNIP2’s anti-inflammatory actions in mice are mediated *via* several downstream effectors, including the NLRP3 inflammasome (30, 31). Therefore, the role of TNIP2 on NLRP3 induced by miR34a was investigated. Testing by Western blotting, it was observed that the protein level of TNIP2 was induced by oxygen, and this induction was blocked by the knockdown of miR34a (Figures 5A,C). TNIP2 mRNA was also elevated by oxygen and inhibited by miR34a knockdown (Figure 5D). Prediction by TargetScan indicated that there existed miR34a binding sites in the 3′ UTR of TNIP2 (Figure 5B), therefore dual-luciferase reporter gene assay was applied to test this prediction. As expected, luciferase activity was greatly reduced with transfection of miR34a and 3’UTR of TNIP2, and was not affected with transfection of mutated miR34a (Figure 5E), which indicated the binding between miR34a and 3’UTR of TNIP2. These above resulted indicated TNIP2 was a potential target of miR34a.

### Overexpression of TNIP2 Inhibited Hyperoxia-Induced Interleukin-1β and Cell Apoptosis

**FIGURE 6:**
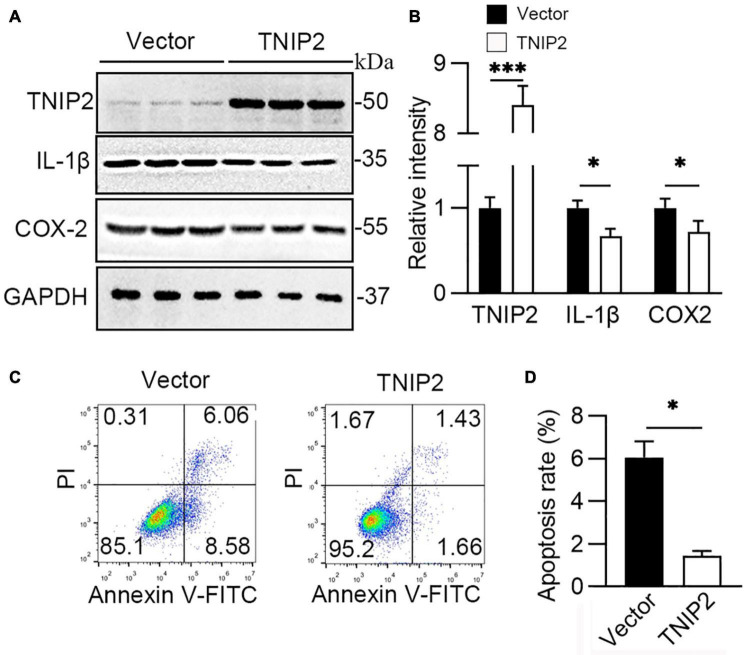
TNIP2 blocks inflammatory pathway and inhibits cell apoptosis induced by hyperoxia. **(A,B)** WT (Vector) and TNIP2 overexpression A549 cells were treated by hyperoxia for 48 h, then analyzed by Western blotting with TNIP2, IL-1β, and COX-2 antibodies and **(B)** their statistical results. **(C,D)** WT and TNIP2 overexpression A549 cells were treated by hyperoxia for 48 h and then analyzed its apoptotic rate using flowcytometry and **(D)** statistical result. Unpaired *t*-test, * *p* < 0.05, ^***^ *p* < 0.001 vs. Vector, *n* = 3.

Given the influence on its expression, the role of TNIP2 on the regulation of lung epithelial cells’ survival and the inflammatory pathway was investigated to demonstrate the mechanistic function of miR-34a. Overexpression of TNIP2 in the A549 cell model revealed a substantial reduction in IL-1β and COX-2 expression induced by hyperoxia (Figures 6A,B). Moreover, flowcytometry analysis revealed that decreasing apoptotic cell in TNIP2 overexpression A549 cells (Figures 6C,D) after induction by hyperoxia compared to vector transfection cells. Thus, it was suggested TNIP2 has a protective role in inhibition of apoptosis induced by hyperoxia.

## Discussion

Preterm infants diagnosed with BPD have an increased risk of abnormal lung function and were characterized mainly by pulmonary inflammation leading to impaired alveolarization and vascular dysregulation (32), and miRNA plays a role in promoting BPD development during this process (33). In this study, it was found that the concentration of proinflammatory cytokine IL-1β in the sputum of infants with ultimately confirmed BPD remain high for a long time after a sharp rise. Then genetic gain-of-function and loss-of-function strategies (including overexpression of miR34a and knockdown of miR34a in A549 cells) were used to provide experimental evidence of the role of miR34a in the production of proinflammation cytokine IL-1β. Furthermore, it was found that TNIP2 was a potential target of miR34a, which may suppress NLRP3 inflammasome activation, IL-1β production, and cell death. The above results indicated the miR34a-TNIP2-IL-1β pathway has a key role in the development of BPD.

With the advancement of therapy and improvement in medical practice, the diagnostic pathological features of BPD have changed over the years. In any case, inflammation is still a major contribution to the pathophysiology of BPD (34). A previous study had demonstrated that in newborn transgenic mice that the expression of IL-1β in the lungs revealed BPD-like characteristics, indicating that IL-1β-induced inflammation in the newborn rat’s lung was sufficient to cause BPD without additional insults (35). Researchers discovered that individuals developing BPD have increased cytokine levels, particularly IL-1β, by using transcriptional profiling to reveal gene modifications in preterm human lung macrophages between health and disease (36). Consistent with these results, it was also a significant increase of IL-1β was observed in the first week after oxygen therapy in those infants who were finally diagnosed with BPD. Other inflammatory cytokines, such as TNF-α, Ang-1, and COX-2, also rose to a greater level when compared to healthy babies, although these cytokines returned to normal by the second week. Only IL-1β concentration maintains a higher level until the fourth week. These suggest that IL-1β has a more profound inducement in the development of BPD. Though IL-1β is mainly produced by alveolar macrophage, alveolar epithelia may play more important role in the progress of BPD as followed: (1) alveolar epithelial type II cells (AECII)-derived chemokine Monocyte chemoattractant protein-1 (MCP-1) was identified as a main factor in activating alveolar macrophages (37); (2) alveolar epithelial type II cells more sensitive to hyperoxia (38). Therefore, it proposed IL-1β secreted by A549 induced by hyperoxia may function as a trigger, recruits and activates alveolar macrophages to secrets more IL-1β, resulting inflammation.

In a multicenter cohort study including infants with gestational age less than 28 weeks, BPD occurred in 3,835 infants of 5,179 infants (74.0%), resulting in a substantial burden on healthcare systems worldwide (39). Therefore, biomarkers or therapeutic targets in BPD diagnostics and prediction appear to be particularly important (40). MiRNAs have been extremely studied in many diseases due to their utility in diagnostics of oncology (41), infectious diseases (42), and lung disease (43). Previous research found that miR34a was considerably increased in the lungs of mice subjected to hyperoxia compared to normal controls, but decreased when exposed to hypoxia (35, 44). Moreover, increased miR-34a has been reported in the lungs of LPS-induced injury and plasma of patients with sepsis who developed shock, indicating a function of miR-34a in the alteration of endothelial homeostasis and inflammation (45). When analyzed *in* vitro hyperoxia-injury A549 cell model, it’s found that the rise of inflammatory factor IL-1β was positively associated with the increase in miR34a. Therefore, we established miR34a overexpression or knockdown cell line to investigate relation between IL-1β and miR34a. Just as we expected, overexpressing miR34a induced higher concentration of IL-1β in hyperoxia-injury A549 cell, while this increase was prevented by miR34a knockdown, suggesting that miR34a was closely related with production of IL-1β. Further, apoptosis induced by hyperoxia was higher when overexpressing miR34a, and inhibited by knockdown. IL-1β, as a key proinflammatory factor, facilitates apoptosis of epithelial cell lines (46). Based on above results, we hypothesis that miR34a was a upstream effector of IL-1β.

Role as a key activator of inflammation, nuclear factor κB (NF-κB) primes the activation of NLRP3-inflammasome and inducing pro-IL-1β and NLRP3 expression (47). TNIP2, also named A20 binding inhibitor of NF-κB activation-2 (ABIN2), was first discovered in a yeast two-hybrid screen, whose function as regulating NF-κB by binding to A20, a well-known anti-inflammatory signaling molecule (48). TNIP2 has been identified as a hub protein in the NF-κB network and interacts both with protein and RNA, suggesting a role in cellular transport machinery, and RNA transcript processing (49). It was reported that a missense variation in the TNIP2 gene was found in two patients with pulmonary arterial hypertension (50) (PAH), indicating TNIP2 also has a role in BPD, as about 25% of infants with moderate to severe BPD would develop BPD-PAH (51). In this study, it was observed that TNIP2 was downregulated markedly after exposure to hyperoxia in A549 cells. Moreover, the knockdown of miR34a inhibited the decrease of TNIP2, and overexpression of miR34a reduced TNIP2 markedly. Prediction by TargetScan revealed that miR34a may bind to the 3’UTR of TNIP2, and this prediction was validated by using dual-luciferase reporter gene method, confirming that TNIP2 was a target of miR34a. Finally, overexpression of TNIP2 in A549 cells could reverse the induction of COX-2, IL-1β, and NRLP3, suggesting TNIP2 has a protective role in the inhibition of the proinflammation pathway. However, as BPD is a multifactorial disease (52) with no clear mechanism of pathogenesis, further study is required.

However, the current study was performed mainly on an *in vitro* cell model, which is difficult to simulate the complex condition of BPD development under physiological conditions. In this study, the A549 cell line was selected for this study due to its human alveolar type II epithelial cell origin. Also, due to the limitations of A549 cells, our next step is to conduct rat animal studies to investigate the development and progression of mir-34a to BPD in premature fetal rats undergoing oxygen and inflammatory exposure. Also, the expression of miR34a was just in A549 cell, but not pulmonary epithelial lining in lungs of infants with BPD, thus we cannot rule out that other miRNAs, such as miR-219-5p, a miRNA increased in BPD infant (53), involved in the production of inflammatory factors.

In conclusion, these findings showed that miR-34a may contribute to infants BPD by enhancing IL-1β through the regulation of the NLRP3-inflammasome pathway. Deleting miR-34a ameliorates the inflammatory response, leading to suppression of A549 apoptosis, which would be activated if miR34a was overexpressed. TNIP2, a downstream of miR34a, negatively regulated NFKB and inhibited the activation of the inflammasome and production of IL-1β, which suggests a potential therapeutic target.

## Data Availability Statement

The original contributions presented in the study are included in the article/supplementary material, further inquiries can be directed to the corresponding author.

## Ethics Statement

The studies involving human participants were reviewed and approved by Ethics Committee of Wuhan Children’s Hospital, Tongji Medical College, Huazhong University of Science and Technology. Written informed consent to participate in this study was provided by the participants’ legal guardian/next of kin.

## Author Contributions

XT and LM performed the experiments. XT and LZ contributed to the data analysis and manuscript drafting. LZ contributed to the design of the experiments and drafting of the manuscript. All authors have read and approved the final version of the manuscript.

## Conflict of Interest

The authors declare that the research was conducted in the absence of any commercial or financial relationships that could be construed as a potential conflict of interest.

## Publisher’s Note

All claims expressed in this article are solely those of the authors and do not necessarily represent those of their affiliated organizations, or those of the publisher, the editors and the reviewers. Any product that may be evaluated in this article, or claim that may be made by its manufacturer, is not guaranteed or endorsed by the publisher.

## Footnotes

1
http://www.targetscan.org/vert_71/
